# Detection of Middlebox-Based Attacks in Healthcare Internet of Things Using Multiple Machine Learning Models

**DOI:** 10.1155/2022/2037954

**Published:** 2022-11-28

**Authors:** Abdulwahid Al Abdulwahid

**Affiliations:** Department of Computer and Information Technology, Jubail Industrial College, Royal Commission for Jubail and Yanbu, Jubail, Saudi Arabia

## Abstract

The huge number of network traffic data, the abundance of available network features, and the diversity of cyber-attack patterns mean that intrusion detection remains difficult even though many earlier efforts have succeeded in building the Internet of Healthcare Things (IoHT). The implementation of an effective algorithm to filter out most of the probable outliers of Round Trip Time (RTT) of packets recorded in the Internet environment is urgently required. Congestion and interference in networks can arise when numerous biosensors in an IoHT system all attempt to communicate at once. Internet of Health Things networks are susceptible to both intra- and internetwork interference. In this research, the Server-Side Includes (SSI) attack is a key issue because it allows for network compromise as part of Internal Attacks. Despite recent advancements, SSI detection remains difficult due to the vast amounts of network traffic data, the abundance of network features, and the diversity of cyber-attack patterns (DDoS, DoS, Satan, spoofing, etc.). With the help of sensors, physiological data may be collected and sent to distant servers, where they can be analyzed in real time by doctors to help them catch diseases in their earliest stages. This is made possible by the Internet of medical things (IoMT). Wireless data transfer, however, leaves it vulnerable to hackers, especially if the data being transferred are particularly private or sensitive. Security measures designed for devices with more storage space and processing power will not work on those with less. However, machine learning for intrusion detection can give a tailored security response to the needs of IoMT systems. For SSI detection, current methods are either inefficient because of the large number of packets that need to be caught and analyzed or unsuccessful because of outlier values in the RTTs obtained from the captured TCP packets. To the same end, “downstream detection” refers to the process of calculating the total length of all connections made after a certain point. As a means of improving the SSI detection algorithm's throughput in a network environment, packet RTT outliers will be eliminated. Flow records are used as inputs by flow-based NIDS to determine whether or not a given flow is malicious. In order to detect middlebox-based attacks from two Medical Health IoT datasets, this paper proposes a unique architecture of explainable neural networks (XNN). The model's accuracy in classifying attacks in dataset 1 of the IoHT is 99.7%t, besides achieving 99.4% accuracy in categorising attacks on IoHT dataset 2.

## 1. Introduction

As IoT technologies continue to advance rapidly, attack methods are getting increasingly sophisticated in their ability to penetrate systems and elude generic signature-based defenses [1]. Machine learning techniques may be a viable option for resolving such complicated and tough problems due to their capacity to quickly adapt to new and unexpected conditions. In computer and information security, various machine learning techniques have been used successfully [2].

New methods to detect and prevent attack traffic from IoT botnets are being developed in response to this expanding risk. It has been shown that machine learning (ML) can be useful for spotting malicious Internet traffic [3]; thanks to recent studies focusing on anomaly detection. Still, there has not been much work done to develop ML models with features tailored to IoT attack traffic or IoT device networks. However, the traffic from IoT devices is typically different from that of other Internet-connected devices (such as laptops and smart phones) [4]. IoT devices, for instance, often only interact with a limited number of endpoints, as opposed to a wide range of web servers.

Also, because of the increased frequency with which IoT devices communicate, network traffic from these gadgets is more likely to exhibit predictable patterns, such as the transmission of brief packets at regular intervals.

Although many previous works have successfully developed Internet of Healthcare [5], intrusion detection is still challenging due to the high volume of network traffic data, numerous available network features, and various cyberattack patterns[6]. Despite this, there have been many previous works that have made some progress. Implementing an effective algorithm that can get rid of the majority of the probable outliers in the round-trip times of packets collected in an Internet environment is an urgent necessity at this point [7]. As a result of the simultaneous communication of a great number of biosensors, there is a potential for network congestion and interference in IoHT. Inter- and intranetwork interference are the two types of network interference that occur most frequently in the Internet of Things (IoT) [3].

As a part of an Internet of Things (IoT) solution, machine learning refers to the ability of an intelligent device to change or automate a knowledge-based state or behaviour. ML algorithms are utilised in tasks like regression and classification because they can infer useful knowledge from data supplied by devices or humans [4]. ML can also be utilised to deliver security services in an IoT network. Employing machine learning in cybersecurity applications is becoming increasingly common, and this trend is expected to continue shortly [8–11]. Many studies have employed ML algorithms to determine the best ways to detect attacks; however, research on efficient detection methods suitable for IoT environments is still restricted in number.

The contributions of this study are as follows:

- Using a recent IoHT dataset, this paper evaluates the performance of various machine learning methods for detecting middlebox attacks in IoT networks.
- Improving the performance of the machine learning algorithm by extracting new features from the dataset and selecting the most applicable features.
- In light of the lack of research on the Bot-IoT dataset, this research can be regarded a potentially major contribution.

## 2. Related Work

Since there is no foolproof method of stopping these attacks, researchers have tried a variety of methods. The nature and severity of attacks are constantly evolving, necessitating the use of novel methods to counter them [12–15]. Standard network analysis approaches are insufficient to ensure the security of network resources, and some researchers have turned to machine learning to learn the various models for attack detection. The NIDS only checks inbound and outbound traffic and does not inspect internal traffic [15–17]. To solve this problem, an intrusion detection and prevention system must be widely deployed across the network. There has been some development in the design of IDSs, but despite this, intrusion detection remains a difficult challenge [6, 18] [19] due to the vast volume of network traffic, the variety of network features available, and the plethora of attacking patterns. It is obvious that false-negative mistakes could happen when using network-based detection algorithms. In order to ascertain the length of the upstream connection, a technique known as “upstream detection” must first be performed. Similar to upstream detection, downstream detection identifies how many links are next in a chain. Because the intruder's host sends Send and Echo packets independently of one another, upstream detection is more difficult and complex [20–22].

The identification of an attacker's Echo packets has no relation to the detection of the attacker's Send packets from the upstream connection. This makes it harder to determine the duration of an upstream link, a persistent problem in SSI detection. If there are no other hosts in the way, the distance between a sensor machine and a target host is essentially the same. It is impossible to detect hostile incursions at this time due to the false-negative errors inherent in network-based detection approaches. If every link in the chain is at least one unit in length and every link is at least two units in length, then the minimum length of the connecting chain is three.

Due to the presence of two downstream connections, it may be concluded that the target host is now under assault and that the session is being manipulated by the attackers. This was the only criterion for the vast majority of network-based detection strategies. Most current network-based detection approaches simply ignore connection chains that are too long to be identified. Conversely, existing network-based SSI detection algorithms are either ineffective or inefficient in the Internet context due to the presence of outlier values in the RTTs produced from intercepted TCP packets.

Intercepted packets will always have RTTs with abnormally high or low values due to the vast variety caused by the intermediary routers in the Internet environment. At first, the authors of this study provide a workable algorithm for removing most of the troublesome RTTs from Internet packets. The authors then employ an improved version of machine learning methods and network traffic mining to develop a reliable SSI detection method. Their proposed SSI detection system for the Internet is said to be precise, efficient, and effective. [23–26]. Flow-based network intrusion detection systems (NIDSs) [27–29] use flow records as an input to determine if a given flow is benign or malicious.

Recently, research has proposed using machine learning (ML) and deep learning (DL) techniques to improve flow-based NIDSs. Positive results have been observed due to the high detection rates achieved by these methods (DRs). It is the author's understanding that the majority of existing solutions rely on the assumption that flow records are derived from a subset of the stream's packets rather than the entire stream itself. For this reason, we have no way of knowing how effectively current ML/DL-based techniques will function in practise. Using a real-world scenario, we examine the impact of sampling, outlier elimination, and packet flow on ML-based NIDS (i.e., when sampling is inevitable).

In order to enhance the Internet system's discriminative capacity and classification performance, the new Deep SDOSVM variant takes into account subclasses within the target class, which is the regular class. The suggested deep SDOSVM method utilises a Dynamic Autoencoder Model (DynAE) for subclasses formation to address limitations in traditional clustering techniques and improve classification performance [30]. It was put through its paces against other state-of-the-art one-class classifiers by being applied to the TON IoT dataset in the real world. Experiments showed the proposed method to be superior to existing related one-class classifiers when applied to network intrusion detection.

A wealth of healthcare records contains information crucial to the continuation of the human race. The analysis of healthcare data is crucial because of the huge potential it has to save lives and improve people's quality of life. The Internet of Things has had a profound impact on modern health care systems and administration (IoT). The IoT is the most promising area for healthcare innovation. This lecture will concentrate on the use of healthcare analytics for the prevention of cardiovascular disease. Recognizing outliers is an essential element of healthcare analytics. The detection of aberrant events in high noise environments helps reduce false-negative alarms (low signal-to-noise ratio). In this example, we will show how smartphone-based cardiac abnormality detection can be used to illustrate the promise of cellphones as a platform for accessible, low-cost m-health [31].

Internet of Medical Things (IoMT) devices, both wearable and nonwearable, are being utilised to improve the accuracy of diagnosis and the speed with which patients can begin receiving treatment for a wide range of conditions. As IoMT devices become more widespread, cybercriminals and other bad actors present a greater risk to human life through actions like data breaches, theft of personally identifiable information, and compromised medical equipment. Data-heavy IoMT devices can keep tabs on your personal and social life, as well as your regular health. Anomalies in this setting may occur as a result of unexpected human behaviour, a faulty sensor, or malicious/compromised device data [32]. Protecting the smart health care infrastructure with a framework that can identify and lessen the impact of abnormalities is essential for addressing this problem. In this research, we introduce an anomaly detection model for RPM that makes use of IoMT and conventional smart home technologies. The authors introduced a hidden Markov model (HMM) based anomaly detection system that analyses regular user behaviour in the context of the RPM, which comprises both smart home and smart health devices. They used information gathered from a variety of IoMT gadgets and home sensors, including information about user networks and behaviour. An anomaly detection approach based on hidden Markov models was devised, and it achieved a 98.6 percent success rate when applied to RPM data.

The Internet of Things (IoT) and its potential applications in healthcare systems are a topic of intense interest to academics. Thanks to IoT innovations, healthcare facilities and patient records may now be tracked and managed in real time. Corporations are creating IoT-based devices with limited data analysis capabilities to compete with one another. In this research, a healthcare system based on the Internet of Things and utilising biomedical sensors was built. This investigation also explores cloud data from biomedical sensors [12] using signal analysis methods for anomaly identification.

In order to keep tabs on patient health and the facility's environment while simultaneously keeping an eye out for network intrusion, an IoT anomaly detection system (ADS) is proposed [33] for usage in smart hospital IoT systems. Having a centralised solution that can track and report on both network performance and EHRs is a huge time saver. Thus, improved choices regarding patient treatment and environmental adaptations may be possible. When data are processed locally, like at the edge, latency is kept to a minimum. The suggested ADS is developed and evaluated with the help of the Contiki Cooja simulator, and the detection of e-health events is based on a study of realistic data sets. The outcomes show a high degree of detection accuracy for both e-health events and IoT network breaches.

The healthcare industry is rapidly adopting IoT solutions to improve efficiency, lower costs, and provide better care to patients. Common components of IoT systems include edge devices such as glucose monitors, ventilators, and pacemakers, gateway devices that aggregate data from the edge devices, and cloud-based systems that analyse the device data to draw conclusions, display information, or direct the connected devices to take action. If this strategy leads to misunderstandings, patient concerns, and treatments may be delayed. The study's [34] focus is on how to leverage Internet of Things (IoT) technology to eliminate these holdups and give patients access to urgent care right away. Wearable device data for patients' health can be monitored and processed using an IoT cloud platform and a model. With the goal of detecting anomalies in patient health data, an offline machine learning model will be constructed and deployed on IoT devices or IoT gateways. Real-time health data will be evaluated locally on the device, with outliers sent to the cloud for further investigation and action.

The medical field's use of the Internet of Things has had a profound impact on patients' lives. Hackers can take over a device and use it to steal information, such as personal health records, or to provide unauthorised access to services. As a result of these limitations, IoT security has been seriously degraded, putting at risk the management of essential infrastructure services. In order to tackle these issues, an anomaly detection of illegal behavior (DIB) system developed for medical IoT contexts is proposed and examined in [10]. The DIB system can learn the rules of operation by analysing data packets from medical IoT devices and it can notify administrators when a device is in an abnormal operating state. They also provided a model using rough set (RS) theory and fuzzy core vector machine to improve DIB anomaly categorization (FCVM). It has been demonstrated that the R-FCVM works well in the lab.

In reference [35], the authors suggest a method that can help healthcare aides in assisted living facilities (ALFs) for people with physical or cognitive handicap carry out their daily responsibilities. This solution bundles together wearable and mobile technologies to improve the quality of support requests and anomaly identification. With the use of this healthcare infrastructure, caregivers can be alerted to any potentially dangerous situations that may arise when residents are out of sight. Plus, no matter where they are in the building, occupants always have access to an emergency call system. There were two types of testing conducted on the system.

With the proliferation of IoT networks in recent years, malicious intrusions attempting to disrupt services and gain access to sensitive patient data have become increasingly widespread. This study demonstrates one approach to improve the safety of networks for medical cyber-physical systems (MCPS) by proposing the creation of new aggregation tiers. Two adversarial neural network (GAN) models trained on the MCPS dataset are provided [36]. Following extensive investigation, scientists concluded that the models developed in the Federated system were superior to those taught in traditional systems when it came to identifying possible security vulnerabilities in a network.

The growing implementation of IoT technology throughout the healthcare industry has led to the development of HealthCare 4.0. In this model, patients' health statuses can be tracked in real-time by RHM software. However, RHM applications frequently experience false alarms. The extreme sensitivity of the monitoring technology, along with genuine variations in the reported vital signs that are unrelated to any impending danger to the patient's health or wellbeing, all contribute to this anomaly. In order to distinguish genuine emergencies from other scenarios, the research presented here [37] employs a wireless body sensor network as its network infrastructure and derives a risk prediction from each piece of sampled data. The experimental results showed an average accuracy and detection rate of 93% and 87.2%, respectively, and the energy consumption profile of the suggested system was found to be compliant with WBSN parameters.

The IoT has given us more leeway in many areas of our lives, such as when dealing with unexpected situations, travelling, managing a building, or receiving medical care. Our study, dubbed wireless body area network (WBAN) [38], focuses on the use of tiny medical sensors. Body-worn sensors like this can record and relay a wide variety of health data. The wireless network makes these apps particularly susceptible to a wide variety of external attacks and anomalies, therefore protecting them is of paramount importance. Jamming attacks can disrupt communication between medical sensors in a WBAN system. This study proposed a novel intrusion detection system (IDS) based on network measurements [39] to distinguish between false alarms caused by jamming conditions and normal state. Our suggested method identifies three types of jamming to lessen false positives and increase detection rates. This IDS method is then simulated using the Castalia platform, which is based on the OMNET++ emulator.

Internet of Things (IoT) advancements in healthcare hold great promise for improving the sector's technological, social, and economic future and thereby ensuring a healthy future for all. Thanks to wireless connectivity between devices in the medical field and the Internet, patients can monitor their health status from afar [40–46]. Real-time patient monitoring, enhanced diagnostic precision, and more efficient treatment are all made feasible by the IoMT. The obvious benefits of these devices should not obscure the fact that they also pose serious privacy and security concerns. Attacks on Internet-connected medical devices could cause major injury or even death to victims. In paper [7], author created a game-changing mobile agent-based intrusion detection system to safeguard the medical device network. It is hierarchical, self-sufficient, and makes use of machine learning and regression algorithms to identify network-level intrusions and anomalies in sensor data. Subsets of IoMT are subjected to extensive testing, such as wireless body area networks and other related medical devices [47–53]. Through simulations, this research demonstrates the potential for achieving high detection accuracy with little resource use.

In recent years, the healthcare business has witnessed dramatic shifts because of the proliferation of IoT devices and the introduction of IoMT technology. The goal of this adjustment is to enhance the comfort of our patients. IoMT networks are vulnerable in a variety of ways because of their heterogeneity and limited resources. Because of their unique characteristics, IoMT networks require novel security approaches, such as highly accurate and efficient anomaly-based intrusion detection systems (AIDSs), to reach their full commercial potential. Anomaly-based intrusion detection (AIDS) was proposed by [39] as a viable security measure for IoMT networks. It is planned to use a combination of host- and network-based technologies to collect logs from IoMT devices and gateways, as well as data from the edge of the network. Despite the computational burden, the proposed AIDS uses machine learning (ML) techniques to spot outliers in the data and, in turn, malicious incidents in the IoMT network.  Table 1 shows the comparative analysis of previous state of art algorithm.

## 3. Proposed Model

**Figure 1:**
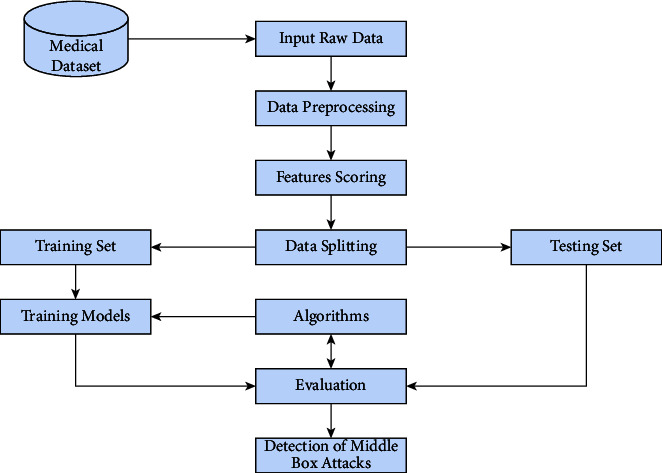
Proposed flow work.

Dataset description, data processing, data cleansing, data preprocessing, feature engineering, model construction with deep learning methods, model performance evaluation, and evaluation of model accuracy are all covered here. The procedure for this study is shown graphically in Figure 1.

Figure 1 illustrates that the CSV file was provided by IoHT DATASET 1 and IoHT DATASET 2. The preprocessing of the data made use of data balance and handling outliers. Cross-validation has been used to ensure the validity of the results. An XNN (explainable neural network) was designed to classify data. This model uses a combination of multilayer perceptron and artificial neural network parameters. In-depth explanations are provided for each of the components.

### 3.1. Dataset

**Figure 2:**
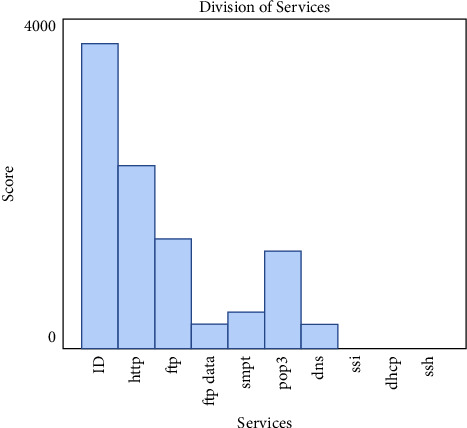
Repartition of services in IoHT DATASET 1 subcategories of accomplishments in the IoHT dataset 1.

IoHT DATASET 1 Dataset records network breaches and can be used to track out the perpetrators. DoS, worms, backdoors, and fuzzers are just a few of the threats present in this nasty spyware. Packets from the network are also included in the Dataset. There are 175,341 records in the training set and 82,332 records in the testing set for attacks and routine attacks. Among the protocols included in the diagram are HTTP, FTP, FTP Data and SMTP, DNS, SNMP, SSL; DHCp, IRC, Radius, and SSH.  Figure 2 shows repartition of services in IOHT DATASET 1 Subcategories of accomplishments in the IoHT DATASET 1.

**Figure 3:**
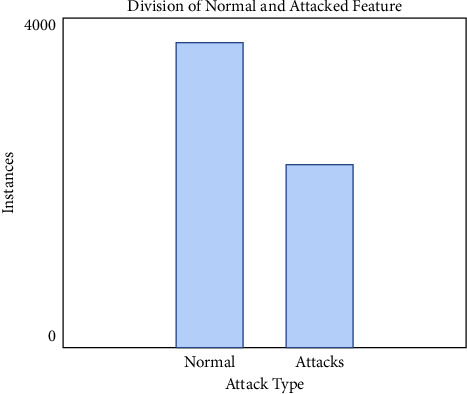
Repartition of attack types.

Datasets include generic, shell code and DOS accomplishments as well as snooping and backdoor achievements. An average of 3500 occurrences per year was found to be within the normal range.  Figure 3 shows repartition of attack types.

Using Kaggle, these data were gathered (an online data source). Unrelated variables and a single related variable are included in the dataset (Outcome).

IoHT DATASET 2 is the second dataset we have used in this project. IoHT DATASET 2 dataset concerns have been addressed by the IoHT DATASET 2 data collection. However, because there are no publicly available datasets for network-based intrusion detection systems, we have that this new version of the IoHT DATASET can still be used as an effective benchmark data set to help researchers compare different intrusion detection methods, despite the problems discussed by McHugh [21].

**Figure 4:**
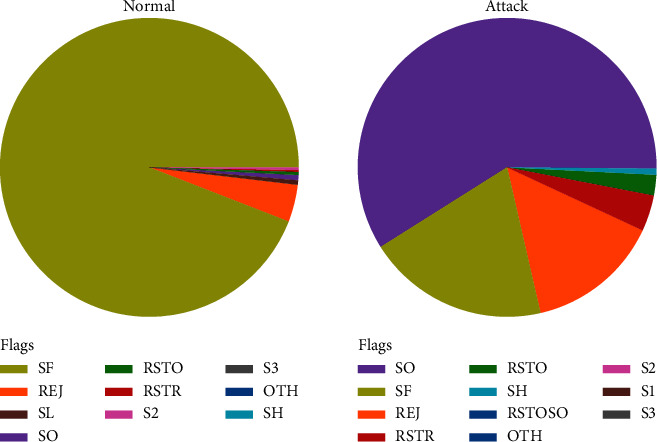
IoHT DATASET 2 visualization.

Training and test sets for IoHT DATASET 2 have enough data. Due to this benefit, studies can be conducted on the complete dataset without the need to randomly select a small portion of the population. Researchers will be able to compare the findings of various investigations as a result of this.  Figure 4 shows IoHT DATASET 2 Visualization.  Table 2 shows the sample distribution.

### 3.2. Raw Data Processing

The unprocessed data were obtained. In the end, a number of methods were used to remove duplicates and null values, among them.

In data mining, this technique is used to turn raw data into a format that can be interpreted. However, in some circumstances, there are discrepancies and/or gaps in the real-world data. Preprocessing procedures include the following:

#### 3.2.1. Data Balancing

Skewed classification is a hindrance to predictive modelling. In most categorization machine learning approaches, each class has the same number of instances. As a result, models from underrepresented groups are underrepresented. When you consider that minorities are more likely to be misclassified than the dominant group, this raises a warning flag. As a result, the study's dataset has been tidied up by eliminating any outliers. These studies have had a considerable impact on the way resampling is done. Under sampling by collecting records from each cluster, for example, can help in conserving information. More varied synthetic samples can be created through over sampling, rather than exact duplicates of minority class data.

#### 3.2.2. Removing of Outliers

We require a well-rounded and homogeneous dataset for doing data mining research. “Outliers” can be found in a dataset. Outliers are values in a dataset that stand out from the norm. A human error, a misreading, or the use of malfunctioning equipment could result in outliers in the data. Before undertaking any statistical analysis or research, it must be removed from the data. Incomplete or erroneous conclusions from data outlines can have an impact on future processing.

When the boxplot data exceed a certain range, the IQR technique is used to eliminate outliers. The interquartile range measures the difference between the upper and lower quartiles (IQR). In order to discover outliers in the data, this study makes use of statistical methods like IQR, Z-Score, and Data Smoothing. The IQR is calculated by taking the 25th and 75th percentiles from a data set and summing them together.

(1) $$\begin{array}{c} IQR=Q3-Q1\text{.} \end{array}$$

#### 3.2.3. Feature Engineering

This is the process of using data from a certain domain to develop functions that may be used by learning machines. It is the process of taking raw data and transforming it into representations suitable for deep learning.

#### 3.2.4. K Means Clustering

It is our goal to make k-means clustering and its variants more understandable by developing a new method for calculating the importance of features. Supervised machine learning makes significant use of the concept of feature importance to make even the most complex models easy to understand. K-Means uses the Euclidean distance metric to account for the difficulties of scaling. Principal component analysis relies heavily on the ability to scale (PCA). Due of the significant variance of high-magnitude features, PCA is biased towards finding the most variable features.

#### 3.2.5. One Hot Encoding

Categorical data variables can be converted to machine and deep learning algorithms via a hot encoding procedure, which increases the accuracy of a model's predictions. Machine learning is prevented from thinking that larger numbers are more significant by using one-hot coding. This does not imply, however, that 8, despite being larger, is of greater importance. No matter how important “laughing” is, it is not more important than “laughing”.

### 3.3. Proposed Classification Algorithms

Neural networks should take the role of machine learning models since they are more efficient (XNNs). With these features and nonlinear modifications learned by the network, anyone may interpret its output in a clear and concise manner (predictions). With the help of this model, researchers may better understand and visualize the relationships between input data and output functions in more complex neural networks. Typical neural networks have a hard time dealing with data that is sequential. System calls are followed by host calls in the IoHT DATASET 1. Normal call sequences and sub-sequences might accompany strange behaviour. As system calls are made sequentially, intrusion detection in IoT must take this into account. Classifying input data in this manner requires that past and current data, as well as their shifted or scaled features, be considered. In order to detect intrusions, *f* (*x*) generates input instances with normal and aberrant sequences, makes adjustments to KMEANS clustered data features to meet the proposed XNN constraints. XNN employs the Additive Index Model, which is:

(2) $$\begin{array}{c} f\left(x\right)=x_{1}\beta_{1}^{Tx}+x_{2}\beta_{2}^{Tx}+x_{3}\beta_{3}^{Tx}+\ldots +x_{k}\beta_{k}^{Tx}\ldots \end{array}$$

**Figure 5:**
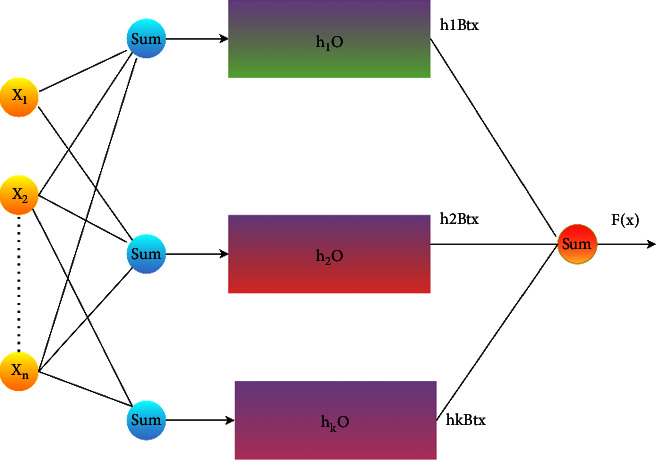
Proposed architecture of XNN.

Adding up the parameters of Shifting, rotating and scaling of data instances, then equation (2) becomes as follows:

(3) $$\begin{array}{c} f\left(x\right)={\mu +x}_{1}\beta_{1}^{Tx}\gamma h_{1}+x_{2}\beta_{2}^{Tx}\gamma h_{2}+x_{3}\beta_{3}^{Tx}\gamma h_{3}+\ldots +x_{k}\beta_{k}^{Tx}\gamma h_{k}\ldots \end{array}$$

where *μ* is the shift parameter used for model fitting and *γ* is the scaling parameter used for fitting as well. The architectural diagram of XNN can be seen in Figure 5:

Data sets in this study can be analyzed with more efficiency when the XNN model has rotating and shifting parameters.

The function F is responsible for classifying output variables like attacks (*x*). Gamma is the input characteristic. K MEANS provides a value based on K Using clustering, so you can keep track of all of your traits in one place. The feature's value is represented by the number *x* in each instance. As Beta increases, so does the scalability coefficient, T. Equation introduces a scaling parameter to the neural network (3). Equation (3) includes the gamma shift parameter with the coefficient of shifting, sigma, and *h* serves as the hyper-parameter transfer function for model over and under fitting.

Weights for each integer in the network are multiplied before they are sent to the next layer of neurons. To arrive at the sigmoid activation function, the weighted sums of each neuron's activation functions must be added up. The weighted connections between layers two and three are now divided by these values. Each subsequent layer is completed in this manner. In a weighted directed network, neurons are represented as nodes, with weighted edges linking them together.

An external environment is fed into a neural network model, which then uses the vectors to store the data. To denote the number of inputs, *x* (*n*) is commonly used. The weights of each input are then added together. In solving a problem, the neural network benefits from the use of weights. The weight of a neural network is frequently used to represent the strength of the connections between neurons in that network. Once all of the inputs have been weighted, total up the weighted sum of all of them (artificial neuron). In order to improve the system's responsiveness, a bias is imposed if the total weighted weighting is zero. The bias is set to “1” for both the weight and the input. Any number from 0 to infinity can be included in the sum. Only if the threshold is sufficiently high can the response match the desired value. An activation function *f* advances the total (*x*). The activation function is activated by transferring control from the transfer function. The activation function might be linear or nonlinear. Below is the pseudocode for neural network.

1. Proce du re Train
2. *X*  ←  training da taset of size *mxn*
3. *y* ←  labels for recor ds in *x*
4. *w*  ← the weight of respective layer
5. *l*  ←  number of layers in the neural network
6. *D*_*i*,*j*_^*l*^  ← The error for all *I*, *j*, *l*
7. *t*_*i*,*j*_^*l*^ ← 0 for all *l*, *I*, *j*
8. For *I*=1 *to* *m*
9. *A*  ←  fee df orwar d(*x*, *w*)
10. *D*(*t*) ←  *a*(*L*)– *y*(*i*)
11. *T*(*I*, *j*) ←  *t*(*I*, *j*)+*a*(*j*). *t*(*t*+1)
12. Else *D*(*I*, *j*) ←  1/*m*(*I*, *j*)

### 3.4. Model Evaluation Parameters

The tactics under consideration were evaluated based on the accuracy, precision, recall, and *F*1 Score criteria. A confusion matrix has been used to show the difference between classed and misclassified clauses.  Table 3 lists the results of the calculations made for each of the metrics considered:

## 4. Results and Discussion

This section summarizes the model's implementation and assessment outcomes. The XNN model was found to be accurate after testing it on both sets of data. In the first step, the study puts the proposed model to be tested against nine attacks from the IoHT DATASET 1. Here, the results of the XNN model and the implementation of the model are shown. Experimentation was carried out using a GPU-based system with Jupyter as the compiler and two 3.2 GHz processors. As a preliminary step, the experiment evaluated the accuracy, precision, recall, and *F*1 of our model's classification of nine attacks from the IoHT DATASET 1 dataset.

### 4.1. Performance of XNN on IoHT DATASET 1 Dataset

**Figure 6:**
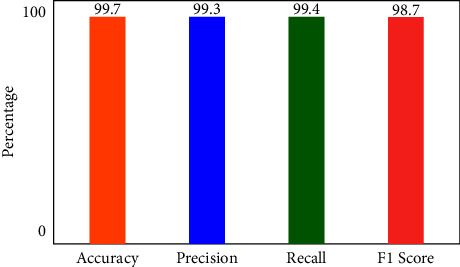
Performance of XNN on IoHT Dataset 1 with KMEANS.

**Figure 7:**
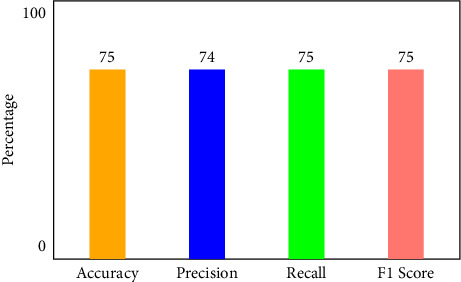
Performance of XNN on IoHT Dataset 1 with one hot encoding.

Figure 6 illustrates that when K-Means-clustering is employed to score features and the XNN model performs well on IoHT DATASET 1. The *y*-axis shows accuracy and the *x*-axis shows precision, recall, and *F*1 scores. In the network-based dataset, the model has an accuracy of 99.7 percent in classifying attacks. When using only one hot encoding method (as illustrated in Figure 7), this model's accuracy drops by 75%.

**Figure 8:**
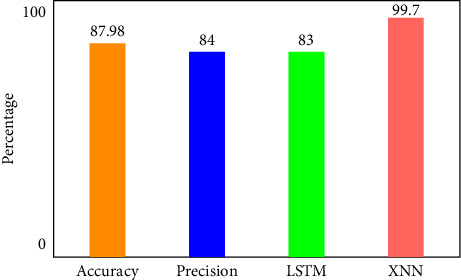
Performance of XNN on IoHT Dataset 1 without feature scoring.

This is lower than the accuracy achieved using feature scoring with KMEANS clustering, which is depicted in Figure 8, despite having a precision of 91.5%.

**Figure 9:**
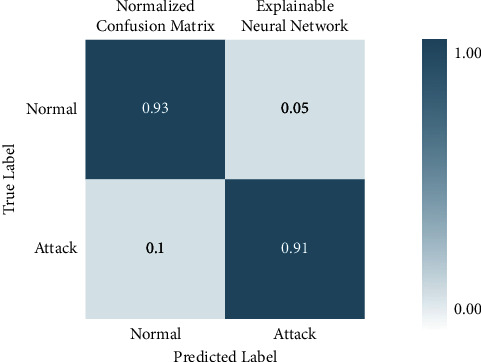
Confusion matrix with KMEANS.

**Figure 10:**
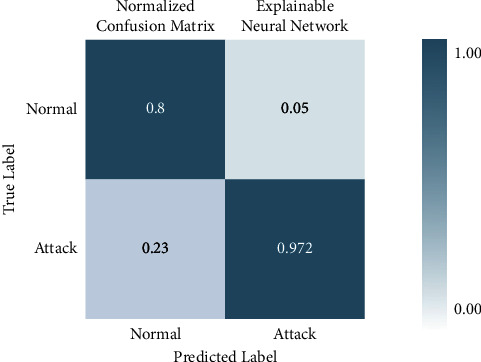
Confusion matrix with one hot encoding.

There are four different axes on the graph: accuracy, precision, recall and *F*1 score. This matrix of confusion is shown in three different ways: with KMEANS, with only one hot encoding, and without feature scoring.  Figure 9 shows how much higher the true positive rate is when KMEANS is used for feature rating. To yet, the most accurate deep-learning model, XNN, has shown promising results.  Figure 10 compares the classification of IoHT DATASET 1 attacks using deep-learning models. The *y*-axis shows the percentage of accuracy, while the *x*-axis shows the model's accuracy histogram.

**Figure 11:**
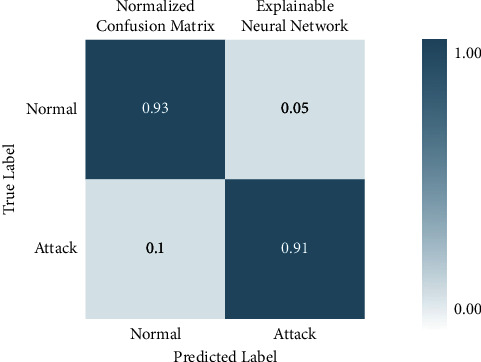
Confusion matrix without feature scoring.

**Figure 12:**
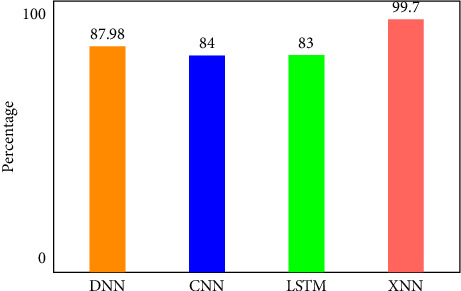
Comparison of deep learning models on IoHT Dataset 1 with KMEANS.

Figure 11 shows the confusion matrix without feature scoring.  Figure 12 shows the comparison of deep learning models on IoHT dataset 1 with KMEANS.

### 4.2. Performance of XNN on IoHT DATASET 2

**Figure 13:**
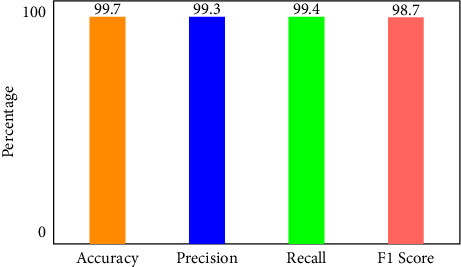
Performance of XNN on IoHT DATASET 2 with KMEANS.

**Figure 14:**
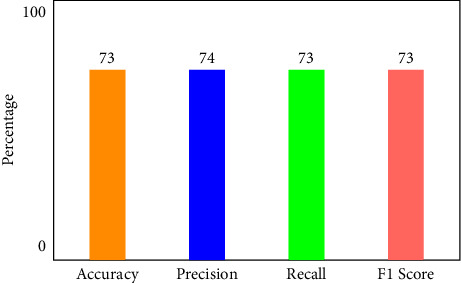
Performance of XNN on IoHT DATASET 2 with one hot encoding.

When K-Means-clustering is employed to score features, as shown in Figure 13, the XNN model does well on IoHT DATASET 2. The *y*-axis shows accuracy, and the *x*-axis shows precision, recall, and *F*1 scores. In the network-based dataset, the model has an accuracy of 99.7 percent in classifying attacks.  Figure 14 shows how inaccurate it is when using just one hot encoding strategy for feature scoring.

**Figure 15:**
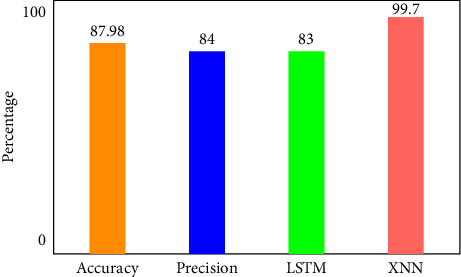
Performance of XNN on IoHT DATASET 2 without feature scoring.

Figure 15 shows that the accuracy of IoHT DATASET 2 maintains 99.7 without feature scoring.

**Figure 16:**
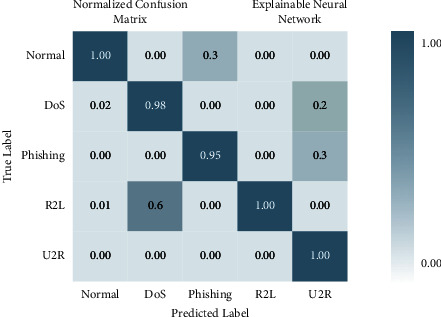
Confusion matrix with KMEANS.

**Figure 17:**
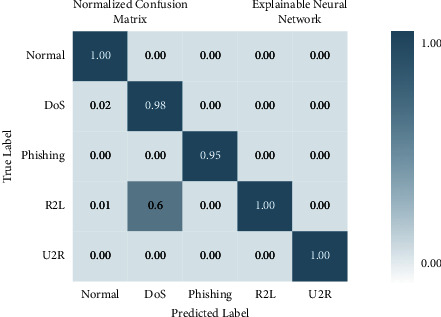
Confusion matrix with one hot encoding.

There are four different axes on the graph: accuracy, precision, recall and *F*1 score. Confusion matrices with KMEANS, One hot encoding, and without feature scoring are depicted in Figures 16 and 17. When KMEANS feature scoring is employed, the true positive rate increases significantly, as seen in Figure 16. Comparison of deep-learning models for classifying attacks is depicted in Figure 17. The *y*-axis shows the percentage of accuracy, while the *x*-axis shows the model's accuracy histogram.

**Figure 18:**
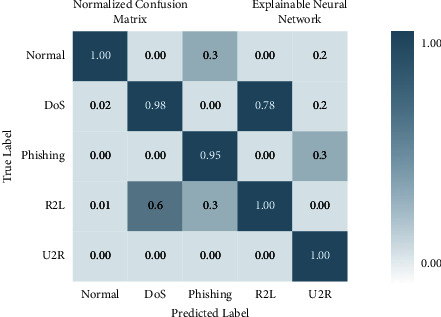
Confusion matrix without feature scoring.

**Figure 19:**
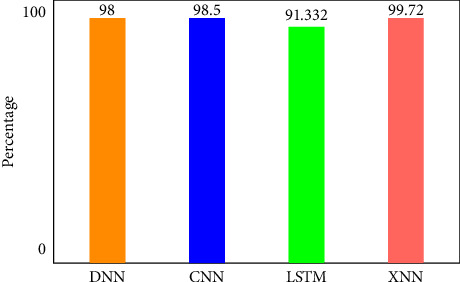
Comparison of deep learning models on IoHT Dataset 2 with KMEANS.

Figure 17 shows the confusion matrix with one hot encoding.  Figure 18 shows the confusion matrix without feature scoring while Figure 19 shows the comparison of deep learning models on IoHT DATASET 2 with KMEANS.

Figure 19 shows the comparison of deep learning models on IoHT DATASET 2 with KMEANS. DNN shows 98% accuracy, CNN shows 98.5% accuracy, LSTM shows 91.332% accuracy and XNN on the highest note shows 99.72% accuracy.

## 5. Conclusions

Intrusion detection is difficult because of the large volumes of network traffic data, the abundance of network characteristics, and the diversity of attacking methods. There needs to be a plan put in place to reduce the number of times when Internet packets have extremely different RTTs. When many IoHT biosensors are all trying to communicate with one another, it can lead to network congestion and interference. Internal and external network interference is a typical issue with the IoHT. It is challenging to detect SSIs due to the enormous amount of network traffic data, the different features of networks, and the complexity of attacker patterns. Low detection accuracy and significant false alarms are the result of out-of-date reference models, ambiguous boundaries between normal and abnormal traffic patterns, and unbalanced data in the face of enormous data volumes. Current SSI detection methods are either inefficient or useless due to outlier RTT values in intercepted TCP packets. The downstream detection technique allows for a preliminary estimation of the downstream connection chain length. By reducing packet RTT outliers, the author has improved the online throughput of the SSI detection algorithm. For detecting malicious flows, NIDS takes flow records as inputs. The author has proposed an XNN architecture for detecting middlebox attacks in Healthcare IoT. (Explainable neural networks). In both experiments, XNN outperformed the baseline models as an efficient technique. In IoHT dataset 1, the model obtains a 99.7 percent accuracy in classifying attacks, whereas in dataset 2, it achieves a 99.4 percent accuracy. To make the system more effective and to help the healthcare sector, it is possible to continue this work on real-time machines and with reinforcement learning in the future.

## Figures and Tables

**Table 1:** Comparative analysis.

Reference	Dataset	IoMT	Technique	Internal attacks	External attacks	Packets flow anomaly	Outcomes	Accuracy (%)	Limitations
Fujita et al. [40]	Real time data	✔	Machine learning	No	✔	✔	Anomaly detection and attacks protection	89	No detection using features
Manimurugan et al. [15]	Sensors data	✔	Machine learning	No	✔	✔	Early attack detection	88.67	No feature scoring
Saheed and Arowolo [6]	Cloud based data	✔	Machine learning	No	✔	✔	Anomaly detection	90	No real-time system
Manimurugan et al. [15]	Sensors data	✔	Machine learning	No	✔	✔	Anomaly detection	91	No detection using features
Aljumaie et al. [41]	Real time data	✔	Machine learning	No	✔	No	Anomaly detection	92	No feature scoring
Meng et al. [3]	Real time data	✔	Deep learning	No	✔	✔	Anomaly detection	91	No real-time system
Ali and Mahmoud [42]	Real time data	✔	Effective NN	No	✔	✔	Anomaly from real-time	89.5	No detection using features
Salem et al. [43]	Sensors data	✔	Efficient NN	No	✔	No	Anomaly from sensors data	92.06	No feature scoring
Sehatbakhsh et al. [16]	Sensors data	✔	Deep learning	No	✔	No	Jamming attacks in WBANS	90	No real-time system

**Table 2:** Sample distribution.

Samples	Training set	Testing set
IoHT DATASET 2	16000	7000
IoHT DATASET 1	22330	1200

**Table 3:** Description of metrics.

Metric	Description
Accuracy	Accuracy=TP/(TP+TN)*∗*100
Confusion matrix	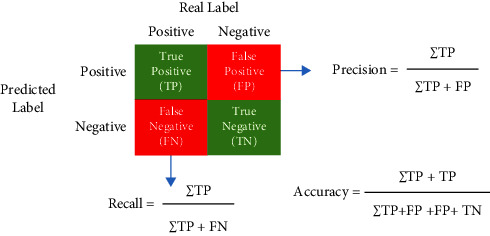

## Data Availability

The datasets used to support the findings of this study are available from the corresponding author upon request.
